# Leptin signaling axis specifically associates with clinical prognosis and is multifunctional in regulating cancer progression

**DOI:** 10.18632/oncotarget.24966

**Published:** 2018-03-30

**Authors:** Tsung-Chieh Lin, Kuan-Wei Huang, Chia-Wei Liu, Yu-Chan Chang, Wei-Ming Lin, Tse-Yen Yang, Michael Hsiao

**Affiliations:** ^1^ Genomic Medicine Core Laboratory, Chang Gung Memorial Hospital, Linkou, Taiwan; ^2^ Genomics Research Center, Academia Sinica, Taipei, Taiwan; ^3^ Department of Diagnostic Radiology, Chang Gung Memorial Hospital, Chiayi Branch, Chang Gung University of Science and Technology, Chiayi, Taiwan; ^4^ Department of Medical Research, China Medical University Hospital, China Medical University, Taichung, Taiwan; ^5^ Department of Biochemistry, College of Medicine, Kaohsiung Medical University, Kaohsiung, Taiwan

**Keywords:** leptin, leptin receptor, expression, survival, prognosis

## Abstract

Leptin is a peptide hormone that has been characterized as the ligand of leptin receptor (*LEPR*). The observation of leptin secretion and leptin receptor expression beyond the normal tissues suggests the potentially critical roles other than its physiological function. In addition to the original function in controlling appetite and energy expenditure, leptin-mediated signaling axis through leptin receptor is multifunctional which plays role in the regulation toward broad types of cancer. Emerging evidences has indicated leptin's function in promoting several processes which are relevant to cancer progression including cell proliferation, metastasis, angiogenesis and drug resistance. We relatively display leptin and leptin receptor expression levels in pan-cancer panel based on the transcriptome analysis via dataset The Cancer Genome Atlas (TCGA), and show the clinical association of the axis in predicting cancer prognosis. The results indicate the pathological impacts of this axis on many types of cancer. This review mainly focuses on leptin-mediated effects and its downstream signaling related to the progression of cancers, and displays the clinical significance of this axis including the impact on cancer patient survival.

## INTRODUCTION

Leptin is the product of *Ob* (*LEP*) gene cloned in1994 by Friedman and colleagues, and was called leptin after the Greek “leptos” meaning thin [1]. Leptin was identified as a peptide hormone released by adipocytes which primarily functions as the ligand of leptin receptor (*LEPR*) to regulate appetite and energy expenditure [2, 3]. The recent discovery of leptin and leptin receptor expression level beyond the traditional tissues indicates that the signaling axis has a critical role outside of its physiological function. The tissues with leptin expression include placenta, stomach, fibroblast, mammary epithelium, and skeletal muscle [4–7]. Furthermore, emerging studies pointed out its expression in broad range of cancer types and also the leptin-dependent signaling in regulating several important factors in cancer progression including tumor proliferation, metastasis, angiogenesis and drug resistance. In this review, we focus on the pathological function of leptin-leptin receptor in cancer, and further illustrate the clinical significance based on the correlation with cancer patient outcomes.

### Leptin and leptin receptor expression in cancer

**Figure 1:**
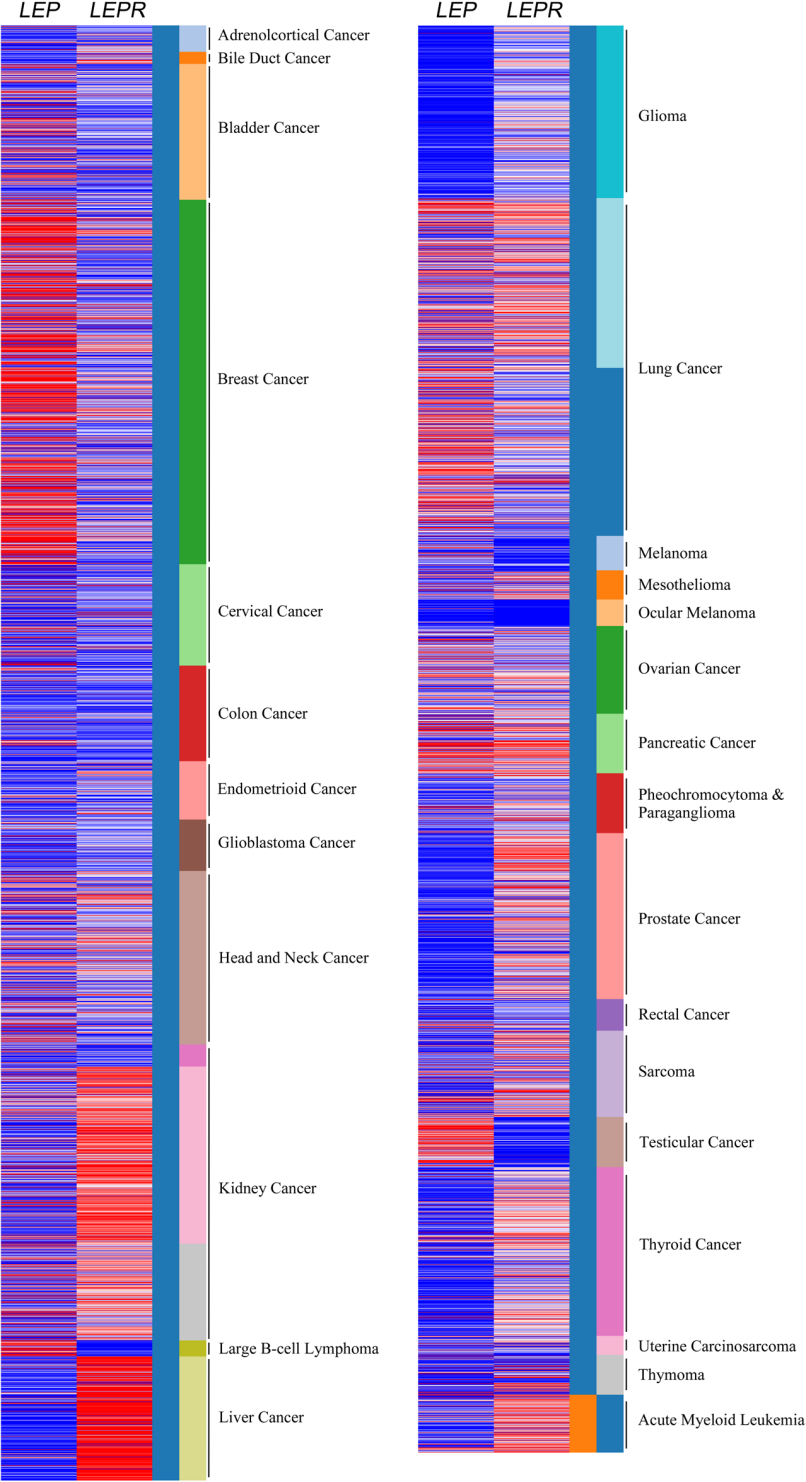
Relative leptin and leptin receptor expression in pan-cancer panel In TCGA pan-cancer dataset, the relative leptin (*LEP*) and leptin receptor (*LEPR*) expression levels were showed in all cancer types. Red color in heat map represents genes with high expression. Blue color in heat map represents gene with low expression.

Cancer cells release leptin and express leptin receptor (*LEPR*), which suggests the potential leptin autocrine/paracrine signaling loop could affect tumor progression. In colorectal carcinoma, relative leptin receptor level displayed the correlation with cancer cell proliferation and neoangiogenesis [8]. The California Santa Cruz (UCSC) Cancer Genomics Browser is a comprehensive analysis tool for understanding the cancer transcriptome data with matched clinical information [9]. The omics data were mainly generated by microarray and RNA sequencing analysis in combination with cancer patient's follow-up data in The Cancer Genome Atlas (TCGA). The results showed the relative leptin and leptin receptor expression in pan-cancer panel (Figure 1). The relative leptin expression level is highly found in bladder cancer, breast cancer, large B cell lymphoma, lung cancer, ovarian cancer, pancreatic cancer, testicular cancer. In addition, leptin receptor is relatively high expressed in kidney cancer, liver cancer, lung cancer, mesothelioma, ovarian cancer, pancreatic cancer, prostate cancer, sarcoma, thyroid cancer and acute myeloid leukemia, indicating the potential pathological role of this axis in cancers.

### The correlation with clinical outcome

The clinical association of leptin or leptin receptor with cancer patient outcome had been explored. The expression of leptin and its receptor were found to be associated with endometrial cancer patient's poorer prognosis (3-year survival rate) [10]. A study in ovarian cancer cohort significantly showed the correlation of leptin and leptin receptor co-expression with shorter patient survival [11]. Furthermore, the increased serum leptin and leptin receptor mRNA expression revealed the positive correlation with cancer recurrence and mortality in triple-negative breast cancer [12]. In addition to the impact of the axis, leptin level alone displayed the positive correlation with poor outcome in ovarian cancer [13], and higher leptin level was found in multiple myeloma patients and associated with clinical stage [14]. Leptin receptor also displayed prognostic power in clinical. Patients of glioblastoma revealed poor prognosis as high leptin receptor levels were detected [15]. The association of leptin receptor with poor recurrence-free survival (*P*=0.09) and cancer-specific survival (*P*=0.01) by log-rank test was observed, and data from Cox regression analysis further characterized leptin receptor as an independent predictor of poor survival respectively with *P*=0.011 and 0.006 in upper tract urothelial carcinomas [16]. However, the observation of negative correlation with cancer progression has also been reported. Cytoplasmic immunohistochemical staining of leptin was less detected in breast cancer with poor survival [17]. In non-metastatic renal cell carcinoma, patients of high disease recurrence rate and poor recurrence-free survival correlated with high CpG methylation in leptin receptor gene [18]. Furthermore, the correlation of leptin and leptin receptor with cancer patient's survival outcome is listed (Table 1 & 2). The expression profile was analyzed by microarray analysis or RNA sequencing technology using samples of cancer patients [9, 19, 20]. The results indicate the potential prognostic power of this axis in predicting the progression of specific cancer type.

**Table 1:** The correlation of leptin with cancer patient survival

Symbol	Cance type	Prognosis	Endpoint	*p* value	Case	Dataset/Cohort	Method	Probe ID
*LEP*	Bladder urothelial carcinoma	-	Overall survival	N.S.	390	TCGA	RNA Seq	
*LEP*	Glioblastoma multiforme	-	Overall survival	N.S.	538	TCGA	RNA Seq	
*LEP*	Breast invasive carcinoma	Poor	Overall survival	0.0013	962	TCGA	RNA Seq	
*LEP*	Cervical squamous cell carcinoma	-	Overall survival	N.S.	191	TCGA	RNA Seq	
*LEP*	Colon and rectum adenocarcinoma	Poor	Overall survival	0.025	467	TCGA	RNA Seq	
*LEP*	Esophageal carcinoma	-	Overall survival	N.S.	184	TCGA	RNA Seq	
*LEP*	Head and neck squamous cell carcinoma	-	Overall survival	N.S.	502	TCGA	RNA Seq	
*LEP*	Acute myeloid leukemia	Poor	Overall survival	1E-05	168	TCGA	RNA Seq	
*LEP*	Kidney pan cancer	-	Overall survival	N.S.	792	TCGA	RNA Seq	
*LEP*	Liver hepatocellular carcinoma	Poor	Overall survival	3E-05	361	TCGA	RNA Seq	
*LEP*	Lung adenocarcinoma	-	Overall survival	N.S.	475	TCGA	RNA Seq	
*LEP*	Ovarian serous cystadenocarcinoma	-	Overall survival	N.S.	578	TCGA	RNA Seq	
*LEP*	Pancreatic adenocarcinoma	-	Overall survival	N.S.	176	TCGA	RNA Seq	
*LEP*	Prostate adenocarcinoma	-	Overall survival	N.S.	497	TCGA	RNA Seq	
*LEP*	Skin cutaneous melanoma	-	Overall survival	N.S.	336	TCGA	RNA Seq	
*LEP*	Stomach adenocarcinoma	-	Overall survival	N.S.	352	TCGA	RNA Seq	
*LEP*	Testicular germ cell tumors	-	Overall survival	N.S.	133	TCGA	RNA Seq	
*LEP*	Thymoma	-	Overall survival	N.S.	118	TCGA	RNA Seq	
*LEP*	Thyroid carcinoma	-	Overall survival	N.S.	489	TCGA	RNA Seq	
*LEP*	Uterine corpus endometrioid carcinoma	-	Overall survival	N.S.	332	TCGA	RNA Seq	
*LEP*	Breast cancer	Good	Overall survival	0.037	1402	E-MTAB-365, E-TABM-43, GSE: 11121, 12093, 12276, 1456, 16391, 16446, 16716, 17705, 17907, 18728, 19615, 20194, 20271, 2034, 20685, 20711, 21653, 2603, 26971, 2990, 31448, 31519, 32646, 3494, 37946, 41998, 42568, 45255, 4611, 5327, 6532, 7390, 9195	Array	207092_at
*LEP*	Gastric cancer	Poor	Overall survival	0.0002	876	GSE: 14210, 15459, 22377, 29272, 51105, 62254	Array	207092_at
*LEP*	Soft tissue cancer	Good	Distant recurrence free survival	0.0006	140	GSE30929	Array	207092_at
*LEP*	Ovarian cancer	Poor	Overall survival	0.0051	278	GSE9891	Array	207092_at
*LEP*	Colon rectal cancer	Poor	Disease specific survival	0.0165	55	GSE17537	Array	207092_at
*LEP*	Brain cancer (Glioma)	Poor	Overall survival	0.046	74	GSE4412-GPL96	Array	207092_at
*LEP*	Breast cancer	Poor	Distant metastasis free survival	0.0489	200	GSE11121	Array	207092_at

Survival data was collected from database SurvExpress, TCGA and Kaplan Meier-plotter.

*LEP*: leptin. N.S.: no significance.

**Table 2:** The correlation of leptin receptor with cancer patient survival

Symbol	Cance type	Prognosis	Endpoint	p value	Case	Dataset/Cohort	Method	Probe ID
*LEPR*	Adrenocortical carcinoma	Good	Overall survival	0.0164	77	TCGA	RNA Seq	
*LEPR*	Cholangiocarcinoma	-	Overall survival	N.S.	35	TCGA	RNA Seq	
*LEPR*	Bladder urothelial carcinoma	-	Overall survival	N.S.	390	TCGA	RNA Seq	
*LEPR*	Glioblastoma multiforme	-	Overall survival	N.S.	538	TCGA	RNA Seq	
*LEPR*	Breast invasive carcinoma	-	Overall survival	N.S.	962	TCGA	RNA Seq	
*LEPR*	Cervical squamous cell carcinoma	Poor	Overall survival	0.0371	191	TCGA	RNA Seq	
*LEPR*	Colon and rectum adenocarcinoma	Poor	Overall survival	0.0309	467	TCGA	RNA Seq	
*LEPR*	Esophageal carcinoma	-	Overall survival	N.S.	184	TCGA	RNA Seq	
*LEPR*	Uveal melanoma	Poor	Overall survival	0.0495	80	TCGA	RNA Seq	
*LEPR*	Head and neck squamous cell carcinoma	-	Overall survival	N.S.	502	TCGA	RNA Seq	
*LEPR*	Acute myeloid leukemia	-	Overall survival	N.S.	168	TCGA	RNA Seq	
*LEPR*	Kidney pan cancer	-	Overall survival	N.S.	792	TCGA	RNA Seq	
*LEPR*	Liver hepatocellular carcinoma	-	Overall survival	N.S.	361	TCGA	RNA Seq	
*LEPR*	Lung adenocarcinoma	-	Overall survival	N.S.	475	TCGA	RNA Seq	
*LEPR*	Ovarian serous cystadenocarcinoma	-	Overall survival	N.S.	578	TCGA	RNA Seq	
*LEPR*	Pancreatic adenocarcinoma	-	Overall survival	N.S.	176	TCGA	RNA Seq	
*LEPR*	Prostate adenocarcinoma	-	Overall survival	N.S.	497	TCGA	RNA Seq	
*LEPR*	Skin cutaneous melanoma	-	Overall survival	N.S.	336	TCGA	RNA Seq	
*LEPR*	Stomach adenocarcinoma	-	Overall survival	N.S.	352	TCGA	RNA Seq	
*LEPR*	Testicular germ cell tumors	-	Overall survival	N.S.	133	TCGA	RNA Seq	
*LEPR*	Thymoma	-	Overall survival	N.S.	118	TCGA	RNA Seq	
*LEPR*	Thyroid carcinoma	Poor	Overall survival	0.0295	489	TCGA	RNA Seq	
*LEPR*	Uterine corpus endometrioid carcinoma	-	Overall survival	N.S.	332	TCGA	RNA Seq	
*LEPR*	Breast cancer	Good	Overall survival	0.051	1402	E-MTAB-365, E-TABM-43, GSE: 11121, 12093, 12276, 1456, 16391, 16446, 16716, 17705, 17907, 18728, 19615, 20194, 20271, 2034, 20685, 20711, 21653, 2603, 26971, 2990, 31448, 31519, 32646, 3494, 37946, 41998, 42568, 45255, 4611, 5327, 6532, 7390, 9195	Array	207255_at
		Good	Overall survival	0.0061				209894_at
		Good	Overall survival	0.053				211354_s_at
		Good	Overall survival	0.0057				211355_x_at
		-	Overall survival	N.S.				211356_x_at
*LEPR*	Ovarian cancer	Poor	Overall survival	8E-05	1656	GSE: 14764, 15622, 18520, 19829, 23554, 26193, 26712, 27651, 30161, 3149, 51373, 63885, 65986, 9891, TCGA (N=565)	Array	209894_at
							RNA Seq	
*LEPR*	Lung cancer	Poor	Overall survival	0.056	1926	CAARRAY, GSE: 14814, 19188, 29013, 30219, 31210, 3141, 31908, 37745, 43580, 4573, 50081, 8894, TCGA (N=133)	Array	207255_at
		Good		3E-11			RNA Seq	209894_at
*LEPR*	Gastric cancer	Poor	Overall survival	1E-06	876	GSE: 14210, 15459, 22377, 29272, 51105, 62254	Array	207255_at
		Poor		0.0024				209894_at
		Poor		0.0004				211354_s_at
		Poor		8E-08				211355_x_at
		Poor		1E-06				211356_x_at
*LEPR*	Colorectal cancer	Poor	Disease free survival	0.0006	55	GSE17537	Array	209894_at
*LEPR*	Acute myeloid leukemia	Poor	Overall survival	0.0029	58	GSE5122	Array	207255_at
*LEPR*	Uveal melanoma	Good	Distant metastasis free survival	0.0099	63	GSE22138	Array	211356_x_at
*LEPR*	Colorectal cancer	Poor	Disease free survival	0.0129	226	GSE14333	Array	209894_at
		Poor		0.0391				211355_x_at
*LEPR*	Colorectal cancer	Poor	Overall survival	0.0139	62	GSE12945	Array	211354_s_at
*LEPR*	Acute myeloid leukemia	Poor	Overall survival	0.0168	79	GSE12417-GPL570	Array	207255_at
*LEPR*	Soft tissue cancer	Good	Distant Recurrence Free Survival	0.0194	140	GSE30929	Array	209894_at
		Good		0.0347				211356_x_at
*LEPR*	Colorectal cancer	Poor	Disease free survival	0.0337	145	GSE17536	Array	209894_at
		Poor		0.0387				211354_s_at

Survival data was collected from database SurvExpress, TCGA and Kaplan Meier-plotter.

*LEPR*: leptin receptor. N.S.: no significance.

### Leptin and cancer cell proliferation

Leptin had been pointed out to regulate cell proliferation particularly in various type of cancer cells. The function in inducing ovarian cancer cell growth was mediated by the increased cyclin D1 and Mcl-1 expression via the activation of the MEK/ERK1/2 and PI3K/Akt signaling pathways [21]. Leptin was reported to promote the increase of esophageal adenocarcinoma OE19 cell proliferation, and the effect was observed to be inhibited by adiponectin receptor axis-mediated suppression of ubiquitin-like with PHD and ring finger domains 1 (UHRF1) [22]. Moreover, leptin alone and co-treatment with secreted phospholipase A2-IIA (sPLA2-IIA) elicited phosphorylation activation of Src/ERK/Akt/mTOR/p70S6K/rS6 pathway leading to increase of 1321N1 human astrocytoma cell proliferation [23]. In breast cancer, low dosage addition of leptin (0.625 nM) significantly induced breast cancer cell growth via increased cell cycle transition. The expression of cell cycle and apoptosis associated factor including p53 and p21WAF1/CIP1 were further proved to be altered [24]. Another study further pointed out the positive correlation between leptin expression and proliferation pathway including leptin receptor, aromatase, mitogen activated protein kinase (MAPK) and signal transducer and activator of transcription-3 (STAT3) in breast cancer patients with estrogen receptor expression and obesity, suggesting the effect of leptin-leptin receptor axis in tumor microenvironment [25]. Similar findings from co-culture system had been reported that the leptin produced by obese adipose stromal/stem cells could enhance proliferation in estrogen receptor positive breast cancers [26]. In addition, the mediator of leptin-leptin receptor in triggering tumor proliferation was identified. APPL1 could directly bind to both STAT3 and leptin receptor to elevate leptin-induced phosphorylation of Akt, ERK1/2 and STAT3 in breast cancer MCF-7 and human hepatocellular carcinoma HepG2 [27], while a reverse function in rat hepatocellular carcinoma proliferation was also reported, this is, via a p38-MAPK-dependent signalling pathway *in vitro* to reduce serum-stimulated H4IIE HCC cell proliferation [28]. In human gallbladder cancer, the experiment result also demonstrated the function of leptin in promoting cell proliferation through leptin receptor (*OB-Rb*), revealing the requirement of leptin receptor in leptin-dependent cancer progression [29]. Clinically, the involvement of leptin receptors expression in proliferation of colorectal carcinoma patients had been observed. Absence in expression level of leptin receptor correlated with low tumor proliferation rate in 94.1% of the cases, while high proliferation rate associated with 92% of the cases with pronounced expression [8].

### Leptin and cancer metastasis

In clinical data, the association of leptin and its receptor expression with bone metastasis was observed in patients of pulmonary adenocarcinoma [30]. In addition, cutaneous melanoma patients with relatively increasing leptin levels in serum samples revealed high risk of sentinel lymph node metastasis [31]. Another correlation analysis also showed the positive association of leptin and leptin receptor with lymph node metastasis in endometrial cancer [10]. In pancreatic cancer, hypoxia inducible factor (HIF)-1α, which associated with patient's metastasis stage and lymph node metastasis, could directly bind to hypoxia-responsive element (HRE) located in *Ob-R* gene promoter (−828/−832) and activated the downstream transcriptional activation, suggesting the potential clinical significance of leptin receptor mediated axis [32]. Actually, recombinant leptin was reported to notably promote ovarian cancer migration, invasion, peritoneal metastasis and epithelial-mesenchymal transition (EMT) via signaling pathway PI3K/Akt/mTOR [13]. Furthermore, TGFβ1 was required for leptin-mediated EMT and metastatic ability in normal epithelial and cancer cell lines of breast (MCF7, MCF10A, MDA-MB-231 and MCF10AT1) [33]. In pancreatic cancer, leptin induced cancer cell migration/invasion and metastasis in orthotopic model, and the simultaneously increased leptin receptor and MMP13 production displayed the positive correlation with patient's TNM stages [34]. Moreover, the studies with regard to anti-leptin-dependent cancer metastasis was explored. Adiponectin, also known as Acrp30, inhibited the leptin-promoted SPEC-2 endometrial cancer metastasis by inactivating JAK/STAT3 pathway via AMPK activation [35]. cAMP elevation also displayed the inhibitory effect to leptin-induced breast cancer MDA-MB-231 migration which was accompanied by a strong decrease of β3 integrin subunit and focal adhesion kinase (FAK) protein levels [36]. The leptin-mediated OVCAR-3 ovarian cancer migration and MMP9 expression could be blocked by 17β-estradiol treatment [37]. Notably, a recent study in colon cancer pointed out that leptin upregulated miR-4443 to repress TRAF4 and NCOA1 expression leading to the decrease cancer invasion [38].

### Leptin and angiogenesis

Leptin receptor appears to be expressed in various types of cancer, suggesting the leptin axis function other than appetite regulation. In human hepatocellular carcinoma, leptin/leptin receptor expressions were detected in both tumor and endothelial cells in parallel with the degree of angiogenesis [39]. In 92% of the colorectal carcinoma cases with pronounced leptin receptor expression, high rate of angiogenesis case was observed alone with the correlation of low grade neoangiogenesis and leptin receptor absence (88.2% of the cases) [8]. In addition, the correlation of leptin receptor with vasculogenic mimicry (VM) formation had been reported in glioblastoma [15]. Moreover, HIF-1α RNA and protein expression of leptin and leptin receptor were high in tissue samples of oral squamous cell carcinoma which indicated the potential crosslink in regulating angiogenesis [40]. Direct evidence had been revealed in the study of human chondrosarcoma cells. Leptin could induce VEGF-C expression and secretion leading to the lymphangiogenesis of human lymphatic endothelial cells via repressing miR-27b [41]. Induction of VEGF-A expression by leptin were found in melanoma tumor [42]. The leptin-mediated regulation toward tube formation of endothelial progenitor cells was also shown in chondrosarcoma cell study. MAPK signaling was activated to enhance AP-1 binding to VEGF-A promoter for transactivation by leptin-leptin receptor axis in cancer cells [43]. Furthermore, HIF-1α and NFκB were proposed as the major route in leptin-dependent VEGF-A induction in breast cancer [44]. The biological significance of leptin-leptin receptor axis in promoting angiogenesis was further shown by the inhibitory effect of Aca 1 treatment, a peptide leptin receptor antagonist, on endothelial cell tube formation [45], as well as by the Allo-aca (leptin receptor antagonist)-mediated reduction of pathological vascularization in rat ophthalmic neoangiogenesis model [46].

### Leptin and drug resistance

Emerging studies referred to the involvement of leptin axis in drug resistance. Leptin levels in serum were higher in patients of pancreatic adenocarcinoma, and associated with gemcitabine chemotherapy [47]. In gastro-oesophageal adenocarcinomas, high leptin expression displayed the resistance to cisplatin, though the effect was not significant to oxaliplatin or 5-fluorouracil [48]. In experimental model of AGS Cis5 and OE33 cell lines, SHLA (leptin receptor antagonist) treatment increased cisplatin sensitivity [48]. The result from another group further pointed out leptin could induce cancer progression and relative molecule expression including ABCB1 protein in pancreatic cancer [49]. Bortezomib-induced toxicity was attenuated accompanying the expression of cyclinD1, Bcl-2 and decreased caspase 3 by leptin addition [14]. Leptin might further regulate stress response and metabolism that cisplatin-induced cytotoxicity in breast tumor cell MCF-7 decreased by leptin alone with SIRT1 upregulation [50]. Similar observation was found that leptin overexpression decreased cisplatin-dependent ER stress unfolded protein response pathways, PERK and ATF6, to induce lung adenocarcinoma A549 cell proliferation [51]. Furthermore, overexpression of leptin receptor led to temozolomide (TMZ) resistance due to the stem/progenitor cell properties, and STAT3 signaling in glioblastoma [52]. Leptin addition attended to block ICI 182,780-mediated therapeutic effects on breast cancer MCF-7 cell proliferation, suggesting the involvement of estrogen signaling axis in drug resistance [53]. However, a survival analysis study indicated the disease-free survival of the tamoxifen-treated postmenopausal obese patients was better in leptin-positive group [54].

### Summary and perspectives

According to the previous findings in publications and *in silico* analysis from the clinical cancer databases, the expressions of leptin and leptin receptor are found in many types of cancer. The signaling axis also plays critical role in regulating several key processes of cancer progression, including cell proliferation, metastasis, angiogenesis and drug resistance. We demonstrated the relative expression level of leptin and leptin receptor in a pan-cancer panel, and revealed the dramatic upregulation of the leptin-leptin receptor axis in breast cancer, head and neck cancer, lung cancer, ovarian cancer and pancreatic cancer, suggesting the potential critical role of this signaling node in tumor progression. The differential RNA expression in specific cancer types suggest the potential alternation in upstream transcriptional activity and RNA stability that might be of value for further investigations in tumorigenesis and cancer progression. In clinical, leptin and leptin receptor serves as poor prognostic markers in variant types of cancer including ovarian cancer, colon cancer, AML, gastric cancer, while the prediction of good outcome by the same axis has also been observed in soft tissue cancer. This might result from potential involvement of other signaling in complicated interaction network contributing to the different outcomes in specific caner type. Obviously, the prognostic function of the axis in breast cancer is currently under debate which might be due to the variances in analytic platform and endpoint that remains to be explored. It is noted the difference in amount of cases enrolled in cohorts also limit the prognostic power, and sufficient patient number for predicting the outcome and reflecting the reality is hard to be determined. Because there have been a few of reports showing discrepancies regarding leptin-leptin receptor axis's function in cancer progression, expression level and clinical outcome, the role of leptin-mediated signaling in promoting or inhibiting cancer progression is still unclear in cancer of specific type. In addition to the variation in the experimental procedures, another possibility is that the leptin-mediated stimulatory or inhibitory effects are partly altered by other receptors in various types of cancer. It is noted that the relative expression levels of leptin and leptin receptor were not uniformly distributed in the pan-cancer cohort, which provides a basis for the pathological involvement of other leptin receptors in tumors, which requires further exploration.
